# Perceived Pregnancy Stress and Quality of Life amongst Iranian Women

**DOI:** 10.5539/gjhs.v6n4p270

**Published:** 2014-04-24

**Authors:** Sara Shishehgar, Mahrokh Dolatian, Hamid Alavi Majd, Maryam Bakhtiary

**Affiliations:** 1 Centre for Cardiovascular & Chronic Care, Faculty of Health, UTS, Tehran, Iran; 2 Nursing and Midwifery Department, Shahid Beheshti University of Medical Sciences, Tehran, Iran; 3 Paramedics Department, Shahid Beheshti University of Medical Sciences, Tehran, Iran

**Keywords:** socioeconomic status, pregnancy, stress, Iran

## Abstract

**Background:**

Stress during pregnancy can result in critical negative outcomes on the mother, the fetus, the newborn, the child and even the adolescent. Quality of life has been recognized as a predictor of stress amongst pregnant women.

**Objectives:**

The first aim of this study was to investigate the role of quality of life in pregnancy stress rates. The second aim was to explore the relationship between maternal stress rate and the four domains of quality of life namely physical health, psychological status, social relationships and environmental conditions.

**Methods:**

The present study was a quantitative cross-sectional research. It was conducted on 210 pregnant women in all trimesters of pregnancy who attended a hospital located in the west of Tehran for prenatal care between August and October 2012. Two questionnaires of The WHO QOL–BREF and Specific Pregnancy Stress were given to respondents to complete. The collected data was analyzed by SPSS version 22 using one-way ANOVA and Spearman correlation and Lisrel 8.8 using statistical path analyzing to describe the direct dependencies among variables.

**Results:**

In the current study, we hypothesized that quality of life may influence the perceived stress during pregnancy. The mean age of the women surveyed was estimated 27±4.8 years. The ultimate result showed that there is a significant relationship between quality of life and pregnancy stress level (Pvalue<0.05, β=-0.16). In addition, we found a significant relationship, as well as direct correlation between the environmental domain in quality of life and the financial and environmental dimensions of specific pregnancy stress (Pvalue<0.05, r=-0.365, r=-0.181).

**Conclusion:**

Further investigations may be considered for extending the results to all pregnant women. Thus, further research across country would be required to validate the results of this study and to generalize the findings to wider population.

## 1. Introduction

Pregnancy has been recognized as a stressful adventure in a productive woman’s life that needs a significant psychological adjustment (Elsenbruch et al., 2007). However, it is often introduced as an exciting time (Talley, 2013). Pregnancy- specific stress is defined as “the imbalance that a pregnant woman feels when she cannot cope with demands, which is expressed both behaviorally and physiologically” (Ruiz & Fullerton, 1999). In addition, “pregnancy-specific anxiety” has been determined as concerns, worries about pregnancy, fear of childbirth, worries about fetus and infant well-being and future parenting (Littleton, Breitkopf, & Berenson, 2007). It is well documented that stress during pregnancy can have an extensive number of maternal as well as neonatal adverse effects. Maternal emotion can influence fetal development and it has clearly been proven in a convergent academic body of evidence (DiPietro, 2012; Talge, Neal, & Glover, 2007; Van den Bergh, Mulder, Mennes, & Glover, 2005). It is well supported by previous studies that pregnancy adverse outcomes and fetal and even neonatal disorders may be results of psychological problems. Maternal anxiety and perceived stress are two negative emotions that induce spontaneous abortion, preterm labor, low birth weight, preeclampsia, suppressed Immune system, vomiting and nausea, raising episiotomy and neonatal infections and negative physical and mental outcomes for both mother and newborn (Bale et al., 2010; Bilbo & Schwarz, 2009; DiPietro, Ghera, Costigan, & Hawkins, 2004; Divney et al., 2012; Rudmin & Ahmadzadeh, 2001; Ruiz & Fullerton, 1999). Moreover, hyperactive school age children and other behavioral issues could be the result of maternal stress during pregnancy.

Additionally, maternal stress has been defined as a most important potential factor that can predict depression during and after pregnancy (Holzman et al., 2006; Lancaster et al., 2010; Records & Rice, 2007; Zelkowitz et al., 2004). It has also attracted researchers’ attention in assessing the relationship of schizophrenia among male offspring with maternal stress during pregnancy (Bale et al., 2010; Khashan et al., 2008; van Os & Selten, 1998).

As a result of the problems mentioned, Public health, families and society could suffer from the short and long-term effects of pregnancy stress (Kingston, Heaman, Fell, Dzakpasu, & Chalmers, 2012). To date, prevalence of pregnancy stress has been reported in a broad spectrum of 33-37% in England and 5-7% in Sweden (Senturk, Abas, Berksun, & Stewart, 2011), While Salari, Firoozi and Sahebi (2005) estimated the prevalence of severe and mild maternal stress 16.7 and 13.6 percent respectively amongst Iranian women.

Three substantial factors including social support, socioeconomic status and quality of life play effectual roles in increasing or decreasing the stress level (Da Costa et al., 2010; Divney et al., 2012; Dunkel Schetter, 2011; Lancaster et al., 2010; Lau & Yin, 2011). Among the three aforementioned factors, the role of quality of life in the perceived stress by pregnant women has been well documented (Lau, 2013; Shishehgar et al., 2013). This means that pregnant women with poorer quality of life experience greater stress during pregnancy rather than their counterparts who enjoy a more desirable quality of life. World Health Organization (WHO, 2009) has defined quality of life as individuals’ perception of their sense of well-being regarding their values, demands and goals. Gordon (2007) pointed out that poor quality of life can result in some adverse symptoms during pregnancy such as heart burn, Nausea and vomiting, legs cramp, as well as dyspnea. She added that these undesirable outcomes can increase the rate of stress among pregnant women with poor quality of life. As Shishehgar et al. (2013) mentioned, quality of life is a measurable variable. Hence, according to its positive impact on pregnancy outcomes with decreasing the perceived stress by pregnant women, it seems that knowledge about mothers’ quality of life is crucial for planning care services for both mothers and their babies. We also found in our previous study in 2013 that quality of life influences the specific-pregnancy stress rate directly. However, that study did not determine how it can increase or decrease the stress rate.

Regarding the importance of the relationship between maternal stress and its adverse outcomes, there is an existing gap in the literature about decreasing or increasing impacts of quality of life on prenatal stress. Therefore, we conducted the present study to provide a conceptualized map to describe the direction of the various domains of quality of life and pregnancy stress dimensions.

## 2. Method and Materials

Conducting this cross-sectional survey was performed between August and October 2012. The number of required participants was calculated as 210 pregnant women regarding to correlation formula. The study population was selected of all pregnant women, according to convenience sampling (which met the study inclusion criteria), who were attending the Shahryar hospital affiliated to Social Security Organization located in Shahryar (a city in the west of Tehran, Iran). Recruiting required samples was done using a purposeful sampling methodology in order to choose pregnant women in different trimesters of pregnancy to gain divergent experiences of stress.

Participants were pregnant women who met the inclusion criteria comprising women in the first or second pregnancy, with a single fetus, without any medical, mental, or disabled spouse or child, without major life event in the last six months, who were non-smokers or drug users and those who have performed the necessary pregnancy care. At the first step, permission from Shahid Beheshti Chancellor and Social Security Organization was granted. Then, participants were informed about the aim of the study and its potential advantages or disadvantages completely. Also, they were assured that in spite of participating in the study they could withdraw any time they wish with the warranty that their information would be kept confidential. Just before commencement the study, they were asked to sign the consent form if they wish to take a part. The privacy of individuals was considered.

Data collected through distinct specific-pregnancy stress and The WHO QOL–BREF questionnaires.

### 2.1 Specific-Pregnancy Stress Questionnaire

Pregnancy stress scale consisted of 51 questions in six domains including health, personal and family, environmental, financial, religion, and how others think about the pregnant mother. This questionnaire was designed in 5-point Likert style from zero (minimum) to 204 (maximum). In all areas grading was performed as follows: 0 = 0, low = 1, medium = 2, high = 3 and very high = 4. Categorizing into three levels was done after the data collection and conversion: mild stress = 0-33.3%, moderate stress = 33.4-66.3%, severe stress = 66.4-100%.

The specific-pregnancy stress scale has been validated by Salari et al. (2005) with test-retest and its reliability has been determined with Cronbach alpha coefficient =0.75.

### 2.2 The WHO QOL–BREF Questionnaire

Quality of life was assessed by a validated Iranian version of the WHO quality of life questionnaire. This comprises 26 items in the four domains of physical health, psychological status, social relationship and environmental condition (WHOQOL-BREF, 1996). It is the short form of the 100 items scale that reflects the multi-dimensional nature of quality of life. Scoring of each question was done according to 5-point Likert style from 1-5. The score of domains was computed according WHO guideline (WHOQOL-BREF, 1996), and total scores were classified into three categories: poor (0-33.3%), average (33.4%-66.3%) and desirable (66.4%-100%). It also has a practical look on quality of life instead of objective life conditions trying to focus on participants’ quality of life (Sajjadi, Vameghi, Ghazinour, & KhodaeiArdakani, 2013). Reliability of this scale was estimated satisfactory using Chronbach’s alpha in four domains (physical health =0.81, psychological status =0.78, social relationships =0.82, and environmental conditions =0.80) (Nedjat, Montazeri, Holakouie, Mohammad, & Majdzadeh, 2008).

### 2.3 Data Analysis

The collected data was analyzed by SPSS.22 using one-way ANOVA to explore the relationship between quality of life and pregnancy stress rate. Then we used Lisrel 8.8 software to analyze data with application of the path analysis. The path analysis method is a way of illustrating the direct and indirect effects of variables in total as well as the relationship between various domains separately. This method of analysis assists us to find out much more about how various items can impact each other.

## 3. Results

This study was conducted on 210 pregnant women with the mean age 27±4.8 years with equal numbers (N=70) in three trimesters of pregnancy. The majority (56.2%) was in the first pregnancy, 93.8% did not experience abortion, 95.7% did not report previous preterm labor, housewives (89%), Azari (40.5%), and had wanted/planned pregnancies (78.6%) (see details in Table 1).

**Table 1:** General information of pregnant women (n=210) attending to Shahryar hospital containing obstetrical variables

Variables	No (%) (N=210)	Mean±SD
**Age, Years**		27±4.8
<25	87(41.4)	
25-30	73(34.8)	
30-35	40(19)	
>35	10(4.8)	
**parity**		
0	*118(56.2)*	
1	92(43.8)	
**Abortion**		
0	197(93.8)	
1	13(6.2)	
**Previous preterm labor**		
0	201(95.7)	
1	9(4.3)	
**Wanted pregnancy**		
Wanted	165(78.6)	
Unwanted	45(21.4)	
**Ethnicity**		
Azari	85(40.5)	
Fars	69(32.9)	
Kord	22(10.5)	
Lor	18(8.5)	
Gilak	16(7.6)	
*Total*	*210*	

Mean scores for quality of life was 64.4±11.3 and for pregnancy stress was 43.6±20.1; It means that our target population has average quality of life and suffers from moderate stress. Using bivariate analyzing a significant and inverse correlation was found between stress and quality of life.

**Figure 1:**
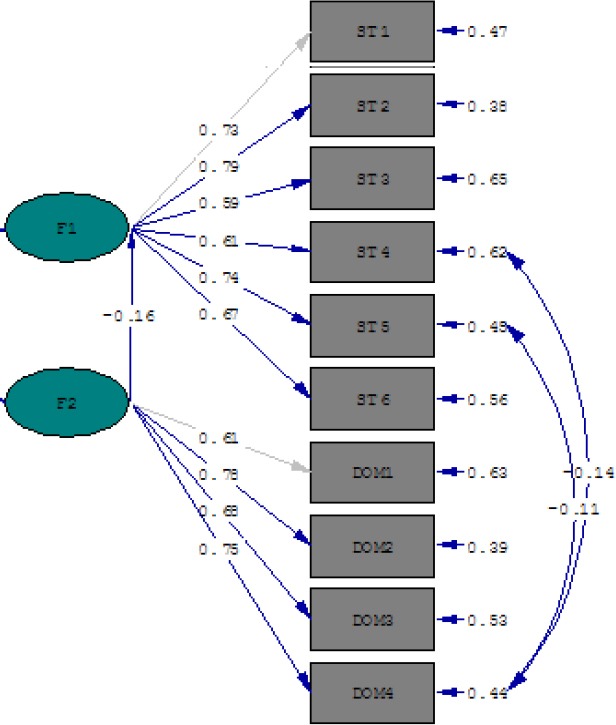
Empirical PATH model for relationship of quality of life (F2) with specific-pregnancy stress (F1) as well as their domains* * ST4= Environmental Dimension, ST5= Financial Dimension, DOM4= Environmental Domain

Also there is a significant relationship between quality of life and stress rate (P<0/001) (see Table 3). In addition, According to the PATH diagram, path of quality of life (β=-0.16) had a significant effect on specific-pregnancy stress rate. The model indicates that desirable quality of life can reduce the stress during pregnancy. As it is obvious in Table 2, only two dimensions of stress including financial and environmental have significant relationship with quality of life. Clarifying this relation, we applied path analysis model to explain how these two variables interact with each other. Figure 1 clearly illustrates that there is a relationship between environmental domain of quality of life and environmental and financial dimensions of specific-pregnancy stress.

**Table 2:** The relationship of quality of life and all six dimensions of specific-pregnancy stress

Variable	Health (Pvalue)	Religion (Pvalue)	Financial (Pvalue)	Environmental (Pvalue)	Personal-Family (Pvalue)	How others think (Pvalue)
Quality of Life	0.346					
	_	0.570				
	_	_	0.001*			
	_	_	_	0.008*		
	_	_	_	—	0.171	
	_	_	_	_	—	0.115

* Quality of life influence pregnancy-stress rate through financial and environmental dimensions

**Table 3:** Relationship and correlation of various stress levels and quality of life during pregnancy

Variables Quality of Life	Mild stress (N=68)	Moderate stress (N=116)	Severe stress (N=26)	ANOVA	Correlation
Poor	0	1(100%)	0	Pvalue=0.026	r= 0.007
Average	28(24.1%)	71(61.2%)	17(14.7%)		
desirable	40(43%)	44(47.3%)	9(9.7%)		
Total	*68*	*116*	*26*		

* Correlation is significant at the 0.05 level

The model fitness was investigated using the indices GFI, CFI and RMSEA (Table 4).

**Table 4:** Goodness of fit indices for the model

	X^2^	df	P GFI	CFI	RMSEA
*Model Index, n=210*	*0.44*	*1*	*0.511*	*1*	*0.000*

Variables indicate that model fits with conceptual model

## 4. Discussion

In this study, path analyzing was used to describe the relationship between theoretical assumption and applied issues of research. Accordingly, quality of life has a direct and significant effect on reducing stress rate amongst pregnant women. A massive body of research documented that quality of life declines during pregnancy (Da Costa et al., 2010; Haas et al., 2005; Mckee, Cunningham, Jankowski, & Zayas, 2001; Tendais, Figueiredo, Mota, & Conde, 2011) due to lower physical activity, limited social functioning and emotional problems (Förger, Oestensen, Schumacher, & Villiger, 2005; Haas et al., 2005; Tendais et al., 2011). As the path model demonstrates, there is a significant and negative correlation between quality of life and stress during pregnancy (Figure 1). We found that poor quality of life can increase the rate of stress. Otherwise, Naito, Sugimoto, Fukuda, Suma and Wakai (2013) pointed out that lifetime stress influences quality of life. They asserted that stressed people have a higher risk of poor quality of life rather than who do not feel stress at all. This reversed finding with ours has been confirmed by Da Costa et al. (2010). They deduced that pregnancy as a stressful time can result in poor quality of life. What can be captured from this study is that physical, emotional and environmental adjustment during pregnancy can result in decreasing quality of life. So, this happening can increase the rate of stress amongst pregnant women and impose heavy emotional and financial burden on families as well as societies.

### 4.1 Strength Points

The current study has some positive points: Firstly, according our knowledge this is a unique study that considered using path model to describe the relationship between quality of life and stress during pregnancy. Secondly, we included pregnant women in all ages of pregnancy in this research. Generally this paper presents a fit model assessing an important variable such as quality of life and its relation with a challenging subject in field of pregnancy and health namely specific-pregnancy stress. Also, it could be a good idea to conduct further researches in different hospitals and maternity centers to be able to extend the results to a wider population.

### 4.2 Limitations

Apart from existent strengths, this study had also some limitations that should be considered for further studies. As a quantitative study, we could not immerse in depth of women’s feeling about their stress and their quality of life. Thus, we offer further research approaching qualitative method (face to face interview, focus group, open-ended questionnaire, semi-structured and in-depth interview) to tackle this hassle. In addition, this study was a cross-sectional survey and the mood of women at the time of completing the questionnaires might influence their responses. It seems to be a good idea to conduct future studies with longitudinal approach to overcome this assumed bias. Finally, the number of questions for assessing stress was too many. Therefore, some participants got exhausted to reply them completely. So, it could be a useful offer for further studies to try to provide a shorter scale that also suit to assess pregnant women’s stress.

In conclusion, this study illustrates that improving the financial condition can influence the level of stress amongst pregnant women. Therefore, it could be a good idea to allocate some financial aids to poor pregnant women and their immediate families and facilitate them by free health care services during and after pregnancy.
